# Supplementary material for: A pH-Controlled Solid Inhibitor Based on PAM Hydrogel for Steel Corrosion Protection in Wide Range pH NaCl Medium
Source: Molecules. 2023 Jan 30;28(3):1314. doi: 10.3390/molecules28031314 (PMC9920852; doi:10.3390/molecules28031314)
Supplement: Supplementary file 1 [file molecules-28-01314-s001.zip › molecules-2153893-supplementary.pdf]

Supplementary material

Figure S1 is the surface and interface pores area distribution of OIM@PAM. The software of "Image J" is employed to count the pores area distribution of OIM@PAM intelligent inhibitor. The area of pores on the OIM@PAM surface and interface is concentrated between 0.02-0.2 μm^2 and 0.02-0.1 μm^2, respectively. The total area of pores on the surface and interface is 14.98 μm^2 and 18.96 μm^2, respectively.

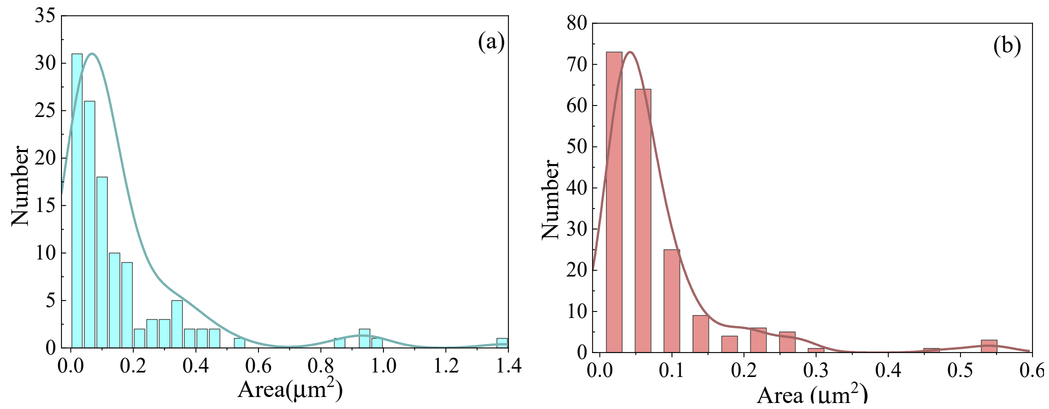

Figure S1. the pores area distribution of OIM@PAM, (a) the surface of OIM@PAM, (b) the interface of OIM@PAM

Figure S2 is the macro morphology of OIM@PAM intelligent inhibitor before and after immersing in different pH solution for168 h. The color and side length change of intelligent inhibitor is analyzed in manuscript.

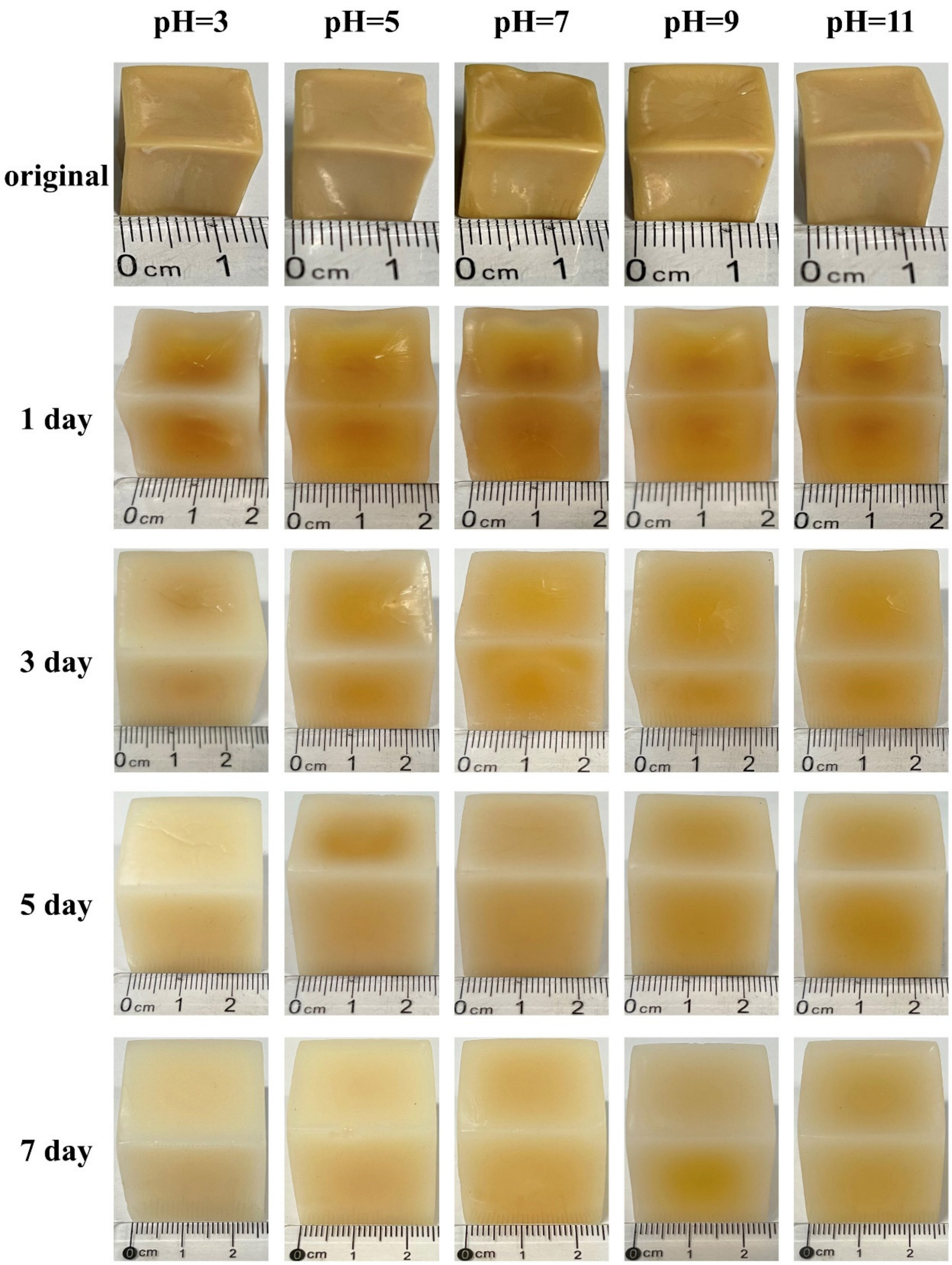

Figure S2 The macro images of as-prepared OIM@PAM immersed in different time in 3.5%(wt) NaCl with virous pH conditions.

Table S1 is the calculated corrosion rate of L80 and the inhibitor efficiency of OIM@PAM in different pH solutions of 3.5% NaCl with and without OIM@PAM at

25 ℃. The long-term anti-corrosion performance and pH-controlled release characteristics is detailly discussed in manuscript.

Table S1 Average corrosion rate and inhibition efficiency of L80 steel in different pH solutions of 3.5%(wt) NaCl without and with OIM@PAM at 25 ℃

pH	Immersion time (h)	Corrosion rate (mm/a)		Inhibition efficiency (%)
		Without OIM@PAM	With OIM@PAM	
3	24	0.7977	0.0551	93.09
	72	0.4124	0.0542	86.86
	120	0.2956	0.0421	85.76
	168	0.2885	0.0434	84.96
5	24	0.6786	0.0665	90.20
	72	0.3675	0.0530	85.57
	120	0.2731	0.0495	81.87
	168	0.2646	0.0512	80.63
7	24	0.3697	0.0434	88.27
	72	0.2790	0.0691	75.24
	120	0.1936	0.0518	73.24
	168	0.1921	0.0539	71.93
9	24	0.3462	0.0292	91.58
	72	0.2484	0.0455	81.78
	120	0.2039	0.0405	80.14
	168	0.1982	0.0464	76.61
11	24	0.3322	0.0255	92.31
	72	0.2612	0.0355	86.39
	120	0.2205	0.0420	80.93
	168	0.2100	0.0437	79.37
